# Lower urinary tract symptoms in men: challenges to early hospital presentation in a resource-poor health system

**DOI:** 10.1186/s12894-020-00651-0

**Published:** 2020-07-03

**Authors:** Ikenna I. Nnabugwu, Ijeoma L. Okoronkwo, Chinwe A. Nnabugwu

**Affiliations:** 1 Department of Surgery, College of Medicine, University of Nigeria Ituku-Ozalla, Enugu, PMB 01129 Nigeria; 2 Department of Health Administration and Management, Faculty of Health Sciences and Technology, University of Nigeria Enugu Campus, Enugu, Nigeria

**Keywords:** LUTS, Men, Medical-care, Challenges, Delay

## Abstract

**Background:**

The point at which men seek medical care for lower urinary tract symptoms (LUTS) varies between individuals. Presentation to hospital with complications beyond LUTS appears prevalent in our setting. The aim of this survey is to assess from the community perspective in southeast Nigeria, the challenges to early presentation for medical evaluation for LUTS by men.

**Methods:**

A questionnaire-based cross-sectional survey of randomly-selected men ≥40 years. The questionnaire captured respondent’s age; presence, duration and severity of LUTS; access to health information; wealth-index; and when (and why) medical care for LUTS was sought. Analysis was with SPSS® version 20.

**Results:**

In all, responses from 1319 men (mean age 54.2 ± 10.2 years) are analysed. Of these, 267 report LUTS: 58.4% (156) report moderate to severe LUTS and 51.7% (138) are yet to seek medical care. As regards seeking medical care, all the men reporting LUTS of 3 months, 35.7% of 126 men reporting moderate LUTS, and 20.0% of 30 men reporting severe LUTS are yet to seek medical care. LUTS being non-bothersome (not financial constraint) is the most prevalent reason for not seeking medical care early. Delay is encouraged by limited access to health information (OR 3.10; *p* < 0.001), but discouraged by literacy (OR 0.86; *p* < 0.001) and aging (OR 0.93; *p* = 0.002).

**Conclusion:**

From the community perspective, the prevalent challenge to seeking medical care for LUTS early is absence of bother. Empowering men through formal education and researched health information will influence positively the time that LUTS in men is appreciated as bothersome.

## Background

Lower urinary tract symptoms (LUTS) are a set of symptoms that usually indicate that there is dysfunction in the storage and controlled evacuation of urine from the urinary bladder [1]. These symptoms can occur in any age group, but the reasons for the symptoms vary from age to age and from one gender to the other [2]. Irrespective of the primary diagnosis, it is suspected that LUTS could have a common pathway [3]. The occurrence of any or some of these symptoms is an indication that there is altered bladder compliance, altered infravesical resistance, increased flow of urine into the bladder, or a combination of these in varying proportions [4]. LUTS may vary from sudden onset transient LUTS, to insidious onset progressive LUTS [5]. In men who are 40 years of age and older, common disease conditions associated with insidious onset progressive LUTS include enlarging benign and malignant prostates, chronic prostatitis, urethral stricture disorders, neoplastic diseases of the urinary bladder, neurogenic bladder disorders, diuresis-causing disorders and many more [6–8]. Some of these disease conditions have attendant grave consequences if not promptly and appropriately addressed medically [9].

Men experiencing LUTS seek medical care at varying points in symptom progression [10]. A number of individual constitutional, social and economic factors, interacting with each other, have been documented to influence the intention to seek medical care: some of them are cultural belief and practice, self-perceived aging [11], literacy level [12], poor finances and accessibility of medical care especially in the absence of robust health insurance [13], and exposure to health information [14].

Left unattended, most of the disease conditions resulting in progressive LUTS and worsening bladder outlet obstruction have increasing risk of life-threatening complications such as obstructive nephropathy, septicaemia, acute urinary retention, and so on [15]. In fact, some of these disease conditions such as prostate and bladder cancers are only curable in the early stages of the diseases, when LUTS alone may be the pointer to the possibility of the diagnosis. Yet allegedly, many men express challenges to appropriate medical care at this early phase of disease evolution [16].

In a typical low-income country with poor health insurance coverage, hospital-based studies report that men diagnosed of benign prostate enlargement (BPE), prostate cancer, bladder cancer, and other disease conditions whose early clinical features are LUTS predominantly present to appropriate hospitals late with complications beyond LUTS [17]. On the other hand, in other climes it has been documented that there is more to seeking medical care for LUTS by men than providing free healthcare services [18]. LUTS of varying severity and duration are considered non-bothersome by men. In addition, men feel embarrassed discussing their LUTS with someone else including medical care providers [19]. The prevalent challenge to early presentation with LUTS in low-income setting may not be financial constraints. The aim of this study therefore is to identify, from the community perspective, the challenges to early presentation to appropriate medical care for LUTS in men. This knowledge will help focus on the right strategies towards improving on early presentation for medical care.

## Methods

This interviewer-assisted, questionnaire-based cross-sectional survey was conducted from March 5th to May 25th, 2018 in Enugu, southeast Nigeria. From the 2006 population figures, Enugu has an estimated population of 983,000 persons, 22.0% of which are men who are 40 years and above [20].

The survey questionnaire which was administered by pre-tutored trainee Urologists and intern doctors, captured respondent’s age and presence or not of LUTS. Where LUTS were reported, the duration of the reported LUTS was ascertained. In addition, the questionnaire assessed respondent’s attitude to seeking medical care early using a 4-point Likert scale on 5 question stems as adapted from Davis et al. [21] The severity of LUTS and the quality of life (QoL) due to the reported LUTS were assessed using validated International Prostate Symptom Score (IPSS) chart [22]. For respondents that had sought medical care for the reported LUTS, the duration of LUTS before medical care was sought as well as the reason for seeking medical care was determined. The nature of medical care sought in the first instance was noted.

Access to possible health information on LUTS was captured in terms of discussions on LUTS with family and peers as well as exposure to conventional media and internet health broadcasts and write ups.

Finally, household living conditions and durable assets adapted from Nigeria-General Household Survey, Panel 2015–2016, Wave 3 [23], were assessed for the purpose of creating respondent’s wealth index. Reliability test yielded Cronbach’s α = 0.735 for the wealth index variables.

The sample size as worked out using the formula $n=\frac{Z^2P\left(1-P\right)}{d^2}$ is 372 subjects; where *P* is 0.591 [24], *Z* is 1.96 and *d* is 0.05. As reported in Nnabugwu et al., (2019) [25] 3 of 9 settlement clusters were randomly selected in Enugu for the study. Within the clusters, the study participants were selected through a systematic random sampling technique of 1 in every 3 eligible men. To be included in the study were men 40 years and older and dwelling within the clusters. Written informed consent was obtained from each eligible participant before inclusion.

Simple frequency was used to determine prevalence of LUTS and nature of medical care sought firstly. Arithmetic mean on compute variable was used to differentiate respondents with good attitude from those with poor attitude to seeking medical care based on responses from the Likert questions focusing on attitude to seeking medical care. Principal Component Analysis (PCA) was used to create wealth indices using the data on household durable assets and living conditions. PCA was also used to create health information indices using responses on possible sources of health information on LUTS. Logistic Regression Analysis was used to evaluate factors that influence duration from onset of LUTS to seeking medical care. Significance was set at *p* < 0.05. All analyses were done with SPSS® version 20. The University of Nigeria Teaching Hospital Bioethics Committee approved of the study.

## Results

**Fig. 1:**
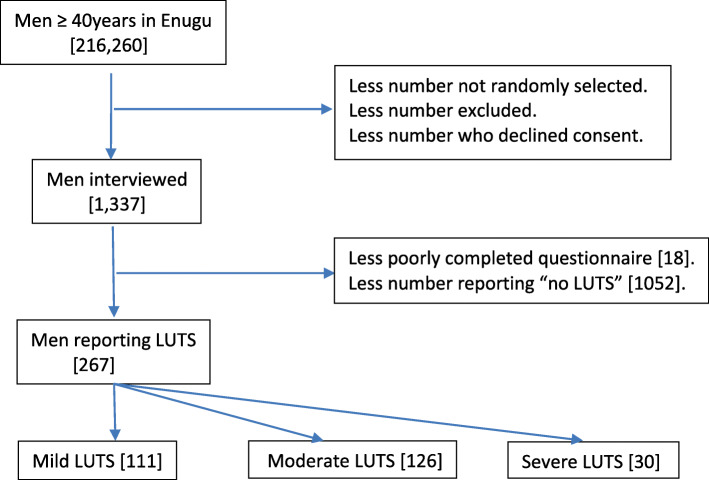
Participants’ recruitment pathway

Figure 1 shows the pathway to patient recruitment in this survey. One thousand three hundred and nineteen (1319) duly completed questionnaires out of 1337 were returned for analysis. The socio-demographic characteristics of these respondents are shown in Table 1.

**Table 1:** Socio-demographic characteristics of respondents

Variables		Frequency (%)
**Age of Respondents (years)**	**40–49**	500 (37.9%)
	**50–59**	420 (31.8%)
	**60–69**	277 (21.0%)
	**70–79**	110 (8.3%)
	**80–89**	10 (0.8%)
	**90–99**	2 (0.2%)
**Mean Age of Respondents (years)**	**54.2 ± 10.2**	
**Duration of Formal Education (years)**	**≤6**	466 (35.3%)
	**>6**	853 (64.7%)
**Mean Duration Formal Education (years)**	**10.5 ± 5.3**	
**Years in Marriage**	**Not Married**	27 (2.1%)
	**≤10 years**	350 (26.5%)
	**11-20 years**	442 (33.5%)
	**> 20 years**	500 (37.9%)
**Number of Siblings**	**None**	14 (1.1%)
	**1–5**	718 (54.4%)
	**> 5**	587 (44.5%)
**Any LUTS**		267 (20.2%)
**Mild LUTS**	**(IPSS ≤ 7)**	111 (8.4%)
**Moderate LUTS**	**(IPSS 8–19)**	126 (9.6%)
**Severe LUTS**	**(IPSS ≥ 20)**	30 (2.3%)

[*LUTS* lower urinary tract symptoms]

Majority of the respondents are between 40 and 59 years of age’ acquired more than 6 years of formal education and reported no lower urinary tract symptoms.

Two hundred and sixty-seven (267) of the 1319 respondents report at least one lower urinary tract symptom which gives overall LUTS prevalence rate of 20.2%. Nocturia at a rate of 19.9% is the most prevalent lower urinary tract symptom. Within the subset of men reporting LUTS (*N* = 267) nocturia is the earliest symptom reportedly noticed (35.2%). The prevalence of LUTS increases with increasing decade of life from 10.2% in the 5th decade of life to 70.0% in the 9th decade. Similarly, the severity of LUTS determined using IPSS worsens with increasing decade of life (χ^2^ 152.9; df 15; *p* < 0.001).

Respondents experiencing LUTS report various durations of LUTS and those who had sought medical care report various durations from onset of symptom(s) to seeking medical care. These are shown in Table 2.

**Table 2:** Duration from onset of LUTS to seeking medical care for the reported LUTS

Duration of LUTS	Yet to seek care	Sought Medical Care		
		Within 3mths	In 4-12mths	After 12mths
**≤3mths (*N*** **= 8)**	8 (100%)	0	0	0
**4-12mths (*N*** **= 51)**	25 (49.0%)	8 (15.7%)	16 (31.4%)	0
**13-24mths (*N*** **= 40)**	20 (50.0%)	1 (2.5%)	11 (27.5%)	8 (20.0%)
**25-60mths (*N*** **= 82)**	47 (57.3%)	1 (1.2%)	15 (18.3%)	19 (23.2%)
**>60mths (*N*** **= 86)**	38 (44.2%)	3 (3.5%)	13 (15.1%)	32 (37.2%)
**Total (267)**	138 (51.7%)	13 (4.9%)	55 (20.6%)	59 (22.1%)

[*mths* months]

Table 2 shows that as symptoms persist or progress, increasing number of men experiencing LUTS reportedly sought medical care.

One hundred and forty-two of the 267 that reported LUTS (53.2%) demonstrate good attitude to early medical care. Eighty-seven of the 111 respondents reporting mild LUTS (78.4%) are yet to seek medical care; 45 of 126 reporting moderate LUTS (35.7%) are yet to seek medical care while 6 of 30 reporting severe LUTS (20.0%) are yet to seek medical care. Twenty-five of 94 respondents (26.6%) reporting symptoms of more than 2 years, which has progressed to moderate to severe LUTS, are yet to seek medical care. Some of the reasons for not seeking medical care yet are shown in Table 3.

**Table 3:** Reasons for not seeking medical care across the various durations of LUTS

Duration of LUTS	Non-Bothersome	Poor Finance	Time Constraint	Advice	Others
**≤3mths (*N*** **= 8)**	4 (50.0%)	2 (25.0%)	2 (25.0%)	0	0
**4-12mths (*N*** **= 25)**	20 (80.0%)	5 (20.0%)	0	0	0
**13-24mths (*N*** **= 20)**	13 (65.0%)	5 (25.0%)	0	0	2 (10.0%)
**25-60mths (*N*** **= 47)**	34 (72.3%)	8 (17.0%)	2 (4.3%)	2 (4.3%)	1 (2.1%)
**>60mths (*N*** **= 38)**	25 (65.8%)	11 (28.9%)	0	2 (5.3%)	0
**Total (138)**	96 (69.6%)	31 (22.5%)	4 (2.9%)	4 (2.9%)	3 (2.2%)

From Table 3, LUTS considered non-bothersome is the most prevalent reason for not seeking medical care for LUTS of various durations.

**Fig. 2:**
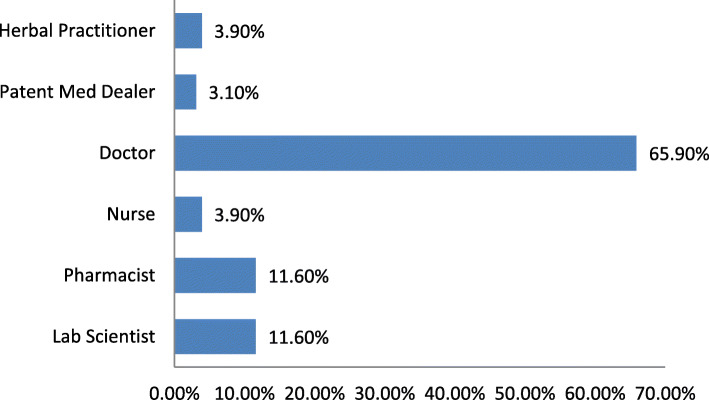
Frequency distribution of care-providers accessed firstly by respondents who had accessed medical care for reported LUTS

On the other hand, of these 267 respondents reporting LUTS, 129 had sought medical care from different care providers. Figure 2 is the frequency distribution of the types of care providers sought in the first instance by these respondents. Some of the reasons for seeking medical care when medical care was sought include: worsening of symptoms (38.0%), persistence of symptoms (26.4%), fear of something worse (18.6%), pressure from family and friends (14.0%) and others including loss of erection (3.1%).

From this figure, most of the respondents that sought medical care reportedly sought the services of orthodox medical doctors firstly.

The variables that influence the decision to seek medical care at the time medical care was sought were subjected to ordinal logistic regression analysis and the result is shown in Table 4.

**Table 4:** Factors that influence the decision to seek medical care for LUTS

Variable	Odds Ratio	95% CI		***p***-value
**Age**	0.93	0.90	0.98	0.002
**Education**	0.86	0.81	0.92	< 0.001
**Marriage**	1.03	1.00	1.06	0.080
**Siblings**	1.00	0.91	1.09	0.929
**Wealth Index 1**	1.60	0.65	3.90	0.303
**Wealth Index 2**	1.41	0.61	3.28	0.425
**Wealth Index 3**	1.15	0.51	2.60	0.743
**Wealth Index 4**	0.87	0.38	1.98	0.740
**Wealth Index 5**				
**Mild LUTS**	6.68	2.62	17.03	< 0.001
**Moderate LUTS**	1.14	0.51	2.55	0.755
**Severe LUTS**				
**LUTS 4-12 months**	0.43	0.22	0.83	0.012
**LUTS 13-24 months**	1.59	0.77	3.29	0.206
**LUTS > 24 months**				
**Low Health Information Index**	3.10	1.79	5.38	< 0.001
**High Health Information Index**				

[*LUTS* lower urinary tract symptoms].

From Table 4, age and formal educational status of respondent, severity and duration of LUTS, as well as health information index of respondent significantly influence the time in the course of LUTS that medical care is sought.

## Discussion

At an overall LUTS prevalence rate of 20.2% and moderate to severe LUTS prevalence rate of 11.9% (Table 1), LUTS in men deserve some emphasis in discussions of men’s health [25]. This is particularly important realizing that 51.7% of all men reporting any lower urinary tract symptom from this survey are yet to seek medical care, and that 32.7% of men reporting moderate to severe symptoms are as well yet to seek medical care. These findings are similar to findings from other studies [24, 26, 27] and are worrisome because of the health implications of seeking medical care late in this health disorder [17]. It is obvious that some of the primary disease conditions resulting in LUTS have poor prognosis with late presentations to medical care [15, 16]. It is therefore quite disturbing that many of these men (Table 2) experience these lower urinary symptoms for years without seeking medical care.

The explanation for this care seeking behaviour in this cohort of men could be that there is some degree of aversion for orthodox medical care generally, or for medical care for LUTS specifically. However, a good proportion (53.2%) of these men reportedly demonstrate good attitude towards early medical care. In addition, when they decided to seek medical care for their LUTS, most (65.9%; Fig. 2) of the respondents from this study consulted medical doctors firstly, demonstrating belief in conventional medical care. So, that these men appreciate the benefit in seeking medical care for health challenges from conventional medical care is not contestable. However, the trigger for seeking medical care for LUTS seems not to be just any lower urinary tract symptom. Curiously, this challenge to early medical care for LUTS seems not peculiar as seen from the review by Roehrborn et al. [28]

From the reasons given by respondents across all LUTS severity and symptom durations for seeking medical care belatedly, LUTS being non-bothersome ranks highest (Table 3) akin to the observation by Griffith et al. [19] So even in low-income economy with poor health insurance coverage, financial constraint does not represent the most prevalent reason for delay in seeking medical care for LUTS in men. To buttress this finding, this study also reveals that respondents’ socio-economic status does not have any significant influence on when medical care is sought by men reporting LUTS (Table 4). It is only rational that one does not commit resources in terms of time and money in accessing care that is of doubtful utility. If LUTS are generally trivialized as part of aging in men, or attributed to some other factors [29], then men will fail to appreciate the utility in accessing medical care for LUTS being experienced. This stance needs to be actively corrected through enlightenment programmes since increasing level of formal education and higher exposure to health information are significantly associated with seeking medical care earlier for LUTS [30]. From this study, there is a 3.1 odd that a respondent of low health information index, due to minimal level of exposure to health information from lifestyle, will seek medical care belatedly compared to a respondent of high health information index (Table 4). Similarly, increase in level of formal education attained is associated with decrease in delay in seeking medical care for LUTS (OR 0.86; *p* < 0.001; Table 4).

As regards age of respondent, the younger the respondent, the longer the delay in seeking medical care (p 0.002). This may be because the younger respondent tends to report milder LUTS, is likely to report less bother, and to attribute higher opportunity cost to seeking medical care. On the contrary, the older respondent is, in addition, likely to be under more pressure from family and peers to seek medical care.

## Conclusion

The observed key challenge with early presentation for medical care by men experiencing LUTS is that LUTS of various severity are considered non-bothersome. Financial constraint comes into play only when LUTS become bothersome. Wealth-index does not appear to influence when LUTS become bothersome, but older age, higher formal education and higher exposure to health information significantly do. Emphasis therefore should be on strategies designed to increase the knowledge base of men on the health implications of LUTS.

The implication therefore, is that empowering men in Nigeria through formal education and improved access to researched health information will influence positively the time that LUTS in men become bothersome. This in turn will help address the problem of late presentation for medical care with complications beyond LUTS.

## Supplementary information

**Additional file 1.**

## Data Availability

The datasets generated and analysed during the current study are available in the Mendeley data repository, [10.17632/dgnnmwt752.1#file-849be15a-04ef-4c7b-ab06-6b1a74f05504].

## Abbreviations

LUTS: Lower urinary tract symptoms
BPE: Benign prostate enlargement
QoL: Quality of life
PCA: Principal component analysis
