# Supplementary material for: Lower urinary tract symptoms in men: challenges to early hospital presentation in a resource-poor health system
Source: BMC Urol. 2020 Jul 3;20:87. doi: 10.1186/s12894-020-00651-0 (PMC7333413; doi:10.1186/s12894-020-00651-0)
Supplement: Supplementary file 1 — Additional file 1. [file 12894_2020_651_MOESM1_ESM.pdf]

QUESTIONNAIRE

Department of Health Administration and Management
Faculty of Health Sciences and Technology
College of Medicine
University of Nigeria Enugu Campus

Dear Sir,

Re: REQUEST FOR YOUR CONSENT TO PARTICIPATE IN THIS CROSS-SECTIONAL SURVEY.

We are medical doctors from University of Nigeria Teaching Hospital, Ituku-Ozalla as well as postgraduate students of the above named department.

We are conducting a research survey titled **"Social and economic determinants of delay in seeking medical care by men 40 years and older with lower urinary tract symptoms (LUTS) in Enugu, Nigeria."**

The objectives of the study include determining the proportion of men 40 years and older who are experiencing lower urinary tract symptoms, identifying the triggers to seeking medical care, characterizing the social and economic factors associated with delay in seeking medical care, and modeling the relationship between these factors and intention to seek medical care when lower urinary tract symptoms begin to manifest.

This survey is essentially for research purposes. The findings from the study will be used to advise policies on health.

To help accomplish the objectives, we request you to give us some of your time and complete this questionnaire for us. The information you provide will be analyzed anonymously. You are free to ask for clarification on any item in the questionnaire and to seek health advice should the need arise in the course of completion of the questionnaire.

If you accept to participate in this study, kindly provide the answers to the questions in the questionnaire, but if you do not accept to participate, kindly return the questionnaire. We respect your decision and we really appreciate your time.

Thank you so much.

Ikenna Nnabugwu
0803 386 6559
(Research coordinator)

Section A: Biodata

1. Age ____

2. Weight _____________________________________

3. Height ______________________________________

4. Mid-abdominal circumference __________________

Section B: International Prostate Symptom Score (IPSS)

5. Do you perceive you have a problem with urination? [Yes] 1 [No] 0

6. The 1st lower urinary tract symptom noticed is? [Freq] 1 [Nocturia] 2 [Urgency] 3 [Hesitancy] 4 [Weak Stream] 5 [Intermittency] 6 Incomplete Emptying] 7

Please complete the IPSS form attached.

Section C: Health Seeking Behaviour (For those that answered* [Yes] *to question 5)

7. How long have you had the problem with urination? [> 5yrs] 5 [2 – 5 yrs] 4 [13 – 24 mths] 3 [7 – 12 mths] 2 [4 – 6 mths] 1 [≤ 3 mths] 0

8. Have you sought medical care for your urinary problem? [Yes] 1 [No] 0

9. **How long from the onset of the urinary symptoms did you seek medical care?** [> 24 mths] 4 [13 – 24 mths] 3 [7 – 12 mths] 2 [4 – 6 mths] 1 [≤ 3 mths] 0

10. Who did you seek medical care from? [Doctor] 4 [Nurse] 3 [Pharmacist] 2 [Lab Scientist] 1 [Others] 0

11. What made you seek medical care for your urinary symptoms?
[Worsening symptoms] 6 [Emergence of another symptom] 5 [Persisting symptoms] 4 [Fear that something worse may happen] 3 [Pressure from family] 2 [Pressure from friends] 1

12. If you have not sought medical care for your urinary symptoms, what may be your reason?
[Poor finances] 5 [No accompanying person] 4 [Symptom not bothersome] 3 [Not sure where to seek medical care] 2 [I was advised not to seek medical care] 1

Section D: Social Support Framework

13. How many years did you spend in formal education? ______________

14. How long have you been married? _____________________________

15. How many siblings do you have? _______________________________

16. How freely do you discuss health-related issues with your family members?
[Very freely] 2 [Not freely] 1 [Not at all] 0

17. Have you discussed your urinary problem with your family? [Yes] 1 [No] 0

18. How freely do you discuss health-related issues with your friends and peers?
[Very freely] 2 [Not freely] 1 [Not at all] 0

19. How do you receive health information from radio or television? [You plan and tune in] 2
[Information come unplanned] 1 [No health information] 0

20. Do you seek health information in newspapers or news magazines? [Yes] 1 [No] 0

21. Do you seek health information in the internet? [Yes] 1 [No] 0

<u>Section E: Socio-Economic Status Variables</u>

22. Your residence is: [Owned by you] 3 [Semi-detached/detached] 2 [Block of flats] 1
[Block of rooms] 0

23. Do you have air-conditioning units in your house/apartment? [Yes] 1 [No] 0

24. What type of power generating set do you have in your house?
[Diesel gen] 3 [Big petrol gen] 2 [Small petrol gen] 1 [None] 0

25. What material is the floor of your house made of? [Tiles/Terrazzo/Rug] 1 [Cement/Mud] 0

26. How many persons sleep in 1 room on average? [1 -2 persons] 1 [≥ 3 persons] 0

27. What energy source do you predominantly use in cooking? [Gas] 2 [Kerosene] 1 [wood] 0

28. Do you have a fridge at home? [Yes] 1 [No] 0

29. Do you have deep freezer at home? [Yes] 1 [No] 0

30. What type of television do you have at home? [Flat screen] 2 [Other types] 1 [None] 0

31. What television broadcast do you subscribe to? [DSTV] 3 [GoTV/Startimes] 2
[Local TV stations] 1 [None] 0

32. Which of these do you own? [Car] 3 [Motorcycle] 2 [Bicycle] 1 [None] 0

33. Does your private car have a functional air-conditioning system? [Yes] 1 [No] 0

34. What schools do(did) your children attend?
[Private/Mission schools] 2 [Public schools] 1 [None] 0

35. Do you have a blender at home? [Yes] 1 [No] 0

Section F: For those that answered [Yes] to question 5

36. Seeking medical care earlier for my urinary problem will be beneficial?

[No] 4 [Unlikely] 3 [Probably] 2 [Yes] 1

37. Seeking medical care earlier for my urinary problem will ensure a better treatment outcome?

[No] 4 [Unlikely] 3 [Probably] 2 [Yes] 1

38. Seeking medical care earlier for my urinary problem will ensure greater value for money spent?

[No] 4 [Unlikely] 3 [Probably] 2 [Yes] 1

39. Seeking medical care earlier for my urinary problem will rub me of my valuable time?

[Yes] 4 [Probably] 3 [Not sure] 2 [No] 1

40. Seeking medical care earlier for my urinary problem makes me feel I am already weakened?

[Certainly] 4 [Probably] 3 [Unlikely] 2 [No] 1

41. My family will prefer I waited for some time before seeking medical care for my urinary problem?

[Certainly] 4 [Probably] 3 [Unlikely] 2 [No] 1

42. My friends will prefer I waited for some time before seeking medical care for my urinary problem?

[Certainly] 4 [Probably] 3 [Unlikely] 2 [No] 1

43. My age mates with similar urinary problems would have sought medical care earlier?

[No] 4 [Unlikely] 3 [Probably] 2 [Yes] 1

44. Activities in my immediate society encourage me to seek medical care for my urinary problem earlier

[No] 4 [Unlikely] 3 [Probably] 2 [Yes] 1

45. I am confident that I have the capacity to seek medical care within 3 months of my urinary problem if I choose to do so.

[Yes] 1 [Probably] 2 [Not sure] 3 [No] 4

46. The decision to seek medical care within 3 months of my urinary problem is entirely up to me

[Agree] 1 [Probably] 2 [Unsure] 3 [Disagree] 4

47. I planned to seek medical care within 3 months of onset of my urinary problems

[False] 4 [Unlikely] 3 [Probably] 2 [True] 1

48. I planned to seek medical care later on if the urinary problem persisted same way

[Yes] 4 [Likely] 3 [Unlikely] 2 [No] 1

49. I planned to seek medical care for my urinary problem when the problem might have worsened

[Agree] 4 [Likely] 3 [Unlikely] 2 [No] 1
